# A Chromosome-Level Genome Assembly for Yamadazyma tenuis ATCC 10573

**DOI:** 10.1128/mra.00213-23

**Published:** 2023-05-25

**Authors:** Lois L. Hoyer, Elizabeth K. Hogan, Soon-Hwan Oh, Brian A. Freeman, Kimberly K. O. Walden, Alvaro G. Hernandez

**Affiliations:** a Department of Pathobiology, College of Veterinary Medicine, University of Illinois Urbana-Champaign, Urbana, Illinois, USA; b Roy J. Carver Biotechnology Center, University of Illinois Urbana-Champaign, Urbana, Illinois, USA; c Department of Mathematics and Computational Sciences, Millikin University, Decatur, Illinois, USA; Vanderbilt University

## Abstract

Pacific Biosciences (PacBio) long-read sequencing was used to generate a chromosome-level genome assembly for Yamadazyma tenuis strain ATCC 10573. The assembly featured 7 chromosomes that matched the electrophoretic karyotype and a 26.5-kb circular mitochondrial genome. The nuclear genome was 10.8 Mb, with a GC content of 43%, and 5,340 predicted genes.

## ANNOUNCEMENT

The genome of Yamadazyma tenuis (formerly Candida tenuis; phylum Ascomycota, class Saccharomycetes, family Debaryomycetaceae) originally was sequenced to understand the comparative genomics of xylose-fermenting yeasts for biofuel production (1). The genome sequence was included in resources such as the *Candida* Gene Order Browser that displays synteny among Saccharomycetes genomes, particularly those associated with human disease (http://cgob.ucd.ie) (2). The availability of long-read DNA sequencing technologies provides the opportunity to improve the *Y. tenuis* sequence and derive a chromosome-level genome assembly that matches the electrophoretic karyotype.

The *Y. tenuis* type strain (ATCC 10573, NRRL Y-1498) was isolated from a bark beetle (USA). The strain was purchased from the American Type Culture Collection (Manassas, VA), grown on yeast extract-peptone-dextrose (YPD) agar (10 g yeast extract, 20 g peptone, and 20 g dextrose, solidified with 20 g Bacto agar per liter), and then stored in YPD liquid medium with 38% glycerol at −80°C. An isolated colony was grown at 25°C in YPD liquid for genomic DNA extraction using a Zymolyase-based protocol (3). Genomic DNA was treated with RNase (Gold Biotechnology) and proteinase K (Gold Biotechnology), and then visualized on an agarose gel to confirm that the DNA was >50 kb.

Genomic DNA was sheared to an average length of 13 kb using a Megaruptor 3 system (Diagenode) and then size selected for fragments of 3 to 50 kb on a BluePippin system with a 0.75% gel cassette and DNA marker S1 (Sage Science). Sheared fragments were converted to a library using the SMRTbell Express Template Prep Kit 2.0 (Pacific Biosciences). The library was sequenced on a single-molecule real-time (SMRT) cell 8M on a PacBio Sequel IIe system using a Sequel II Binding Kit 2.2, the circular consensus sequencing (CCS) mode, and a 30-h movie time. CCS and demultiplexing analysis used SMRT Link v10.0 (ccs –min-passes 3 –min-rq 0.99; lima –hifi-preset SYMMETRIC –split-bam-named –peek-guess). The data set included 634,178 reads (13,092-bp mean read length).

Filtlong v0.1.2 (4) selected a sequence read subset (15-kb minimum length) that represented 50× coverage of the expected 11-Mb genome (1). The genome assembly was produced using hifiasm v0.16.1 with default parameters (5). Primary contigs (i.e., not haplotigs) were deposited into the NCBI database and used in genome assembly assessment and annotation.

**FIG 1:**
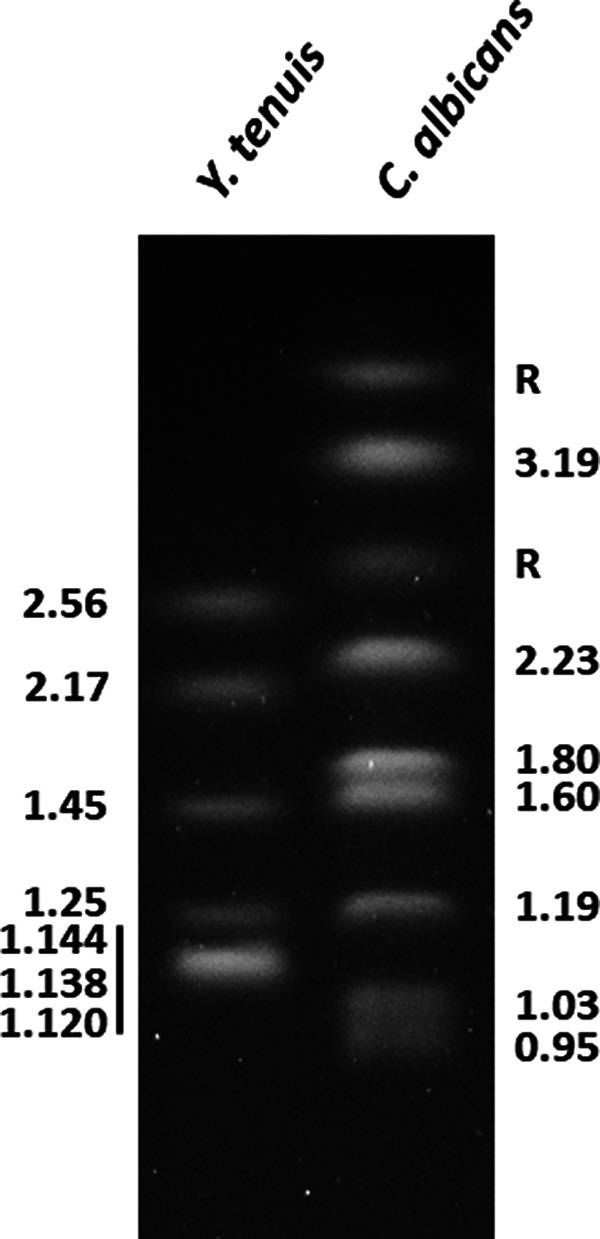
Ethidium bromide-stained chromosomes from *Y. tenuis* (left) and Candida albicans (right); both are diploid species. The well-known C. albicans karyotype provided size standards (17). Chromosomes from each yeast species were embedded in agarose and separated by clamped homogeneous electric field electrophoresis (18). Chromosome sizes (in Mb) were placed at the sides of the image. In C. albicans, chromosome R size is variable due to expansion and contraction of ribosomal DNA (rDNA) tandemly repeated units (17). Moreover, chromosome R homologs may vary considerably in size as observed here. The smallest *Y. tenuis* band represented the comigration of three pairs of similar-sized chromosomes (vertical bar), consistent with its intense staining.

The assembly included 8 gapless contigs. One was a circular mitochondrial genome of 26,498 bp. Sizes of the other 7 contigs matched the electrophoretic karyotype for the *Y. tenuis* strain (Fig. 1). Except one end of chromosome 2, chromosomal contigs had terminal consensus repeats (5′-GTATGGGTCAGAACTTCGGTGGGT-3′) consistent with telomeric sequences in budding yeasts that were described previously (6). The completeness of the genome assembly was evaluated using BUSCO v5.3.2 (7). The percent complete and single-copy BUSCOs were 96.0% (fungi_odb10), 94.5% (ascomycota_odb10), and 99.1% (saccharomycetes_odb10).

Transcriptome sequencing (RNA-Seq) data were collected to inform gene model prediction. *Y. tenuis* ATCC 10573 was grown at 30°C and 200 rpm shaking in synthetic complete (SC) medium (8) with 2% glucose (18 h and 48 h), SC medium with 2% xylose (18 h and 48 h), and YPD medium (6 h and 18 h). SC medium used yeast nitrogen base without amino acids and without ammonium sulfate; a total of 1 g/L ammonium sulfate was added and the final medium sterilized by filtration. Cells were collected by centrifugation, washed once with sterile deionized water, and then flash frozen in dry ice/ethanol and stored at −80°C. RNA was extracted using a hot-phenol method (9). RNA-Seq libraries were prepared using the KAPA mRNA HyperPrep Kit (Roche; catalog number KK8581) to produce stranded reads. The libraries were pooled and quantitated by PCR. The libraries were sequenced on one SP lane for 251 cycles from each end of the cDNA fragments on a NovaSeq 6000 instrument (Illumina). Over 172 million reads were recorded for an average of approximately 14 million stranded reads per sample. Fastq files were generated and demultiplexed with the bcl2fastq v2.20 conversion software (Illumina). Adaptors were trimmed from the 3′ end of each read. Trinity v2.14.0 (10) was used to produce a transcriptome assembly (--SS_lib_type RF).

Next, funannotate v1.8.13 (11) was used to clean, sort, and mask the hifiasm nuclear genome assembly using default parameters. Funannotate predict was aided by the Trinity transcriptome assembly data. EMBOSS Transeq (12, 13) was used to translate coding sequences in the predict output file with Translation Table 12. Table 12-predicted protein sequences were analyzed with InterProScan v5.56-89.0 (14) and eggNOG-mapper web v2.1.9 (15, 16). The output files were passed to funannotate annotate.

The new genome assembly (GCA_029203305.1) predicted 5,430 genes, which is approximately 200 fewer than the representative genome sequence (GCA_000223465.1) (Table 1). The new assembly decreased the contig number by approximately 10-fold and closed 49 gaps.

**TABLE 1:** Comparison of statistics for two *Y. tenuis* genome assemblies

Feature	GCA_000223465.1	GCA_029203305.1
Total ungapped length (bp)	10,582,270	10,831,699
No. of contigs	74	7
Contig *N*_50_ (bp)	382,504	1,446,176
No. of scaffolds	25	7
Size of the largest scaffold (bp)	2,534,490	2,557,709
Scaffold *N*_50_ (bp)	1,222,892	1,446,176
No. of spanned gaps	49	0
GC content (%)	43	43
Sequencing coverage (×)	27	50^*a*^
No. of genes	5,533	5,340

a PacBio sequence reads provided 766× coverage of the *Y. tenuis* genome. The GCA_029203305.1 assembly was created using filtlong v0.1.2 (4) to select reads with a minimum length of 15 kb that represented 50× genome coverage.

### Data availability.

This whole-genome sequencing project was deposited in GenBank (PRJNA932435). The genome assembly (ASM2920330v1) was given accession number GCA_029203305.1. Accession numbers CP117537.1 to CP117544.1 were assigned to the chromosomes and mitochondrial genome sequence described here. Sequence reads were deposited in the Sequence Read Archive (SRR23370013). The RNA-Seq data set was deposited under accession number SRR23370326. The project was assigned the locus prefix tag PSN45 (SAMN33192236).
